# Identify a DNA Damage Repair Gene Signature for Predicting Prognosis and Immunotherapy Response in Cervical Squamous Cell Carcinoma

**DOI:** 10.1155/2022/8736575

**Published:** 2022-03-23

**Authors:** Hong Zhou, Limei Wu, Lijun Yu, Yabing Yang, Lili Kong, Shuo Liu, Wenhui Chen, Ruiman Li

**Affiliations:** ^1^Department of Obstetrics and Gynecology, The First Affiliated Hospital of Jinan University, Guangzhou 510632, China; ^2^Department of Oncology, The First Affiliated Hospital of Jinan University, Guangzhou 510632, China; ^3^Department of Gynecology, Beijing Rehabilitation Hospital Capital Medical University, Beijing, China; ^4^Department of General Surgery, The First Affiliated Hospital of Jinan University, Guangzhou 510632, China

## Abstract

The DNA damage repair (DDR) genes are increasingly gaining attention as potential therapeutic targets in cancers. In this study, we identified the DDR genes associated with the tumor mutation burden (TMB) and prognosis of cervical squamous cell carcinoma (CESC) based on The Cancer Genome Atlas (TCGA) database. Through LASSO Cox regression, the prognostic signature involving five DDR genes (ACTR2, TEX12, UBE2V1, HSF1, and FBXO6) was established, and the risk score was identified as an independent risk factor for CESC. The nomogram consisting of the five genes accurately predicted the overall survival (OS) and the immunotherapeutic response of CESC patients. Finally, the loss of the copies of the transcription factor (TF) SP140 in CESC patients may decrease the expression of FBXO6, improve DNA repair function, and reduce the diversity of neoantigens, thereby lowering the response to immunotherapies. Therefore, the DDR gene signature is a novel prognostic model and a biomarker for immunotherapies in CESC patients.

## 1. Background

With 530,000 newly diagnosed cases each year, cervical squamous cell carcinoma (CESC) is the fourth most common cancer worldwide and the third most common cancer in women [1, 2]. Almost all CESC cases are the result of human papillomavirus (HPV) infection [3]. While cervical screening and antiHPV vaccination are effective preventive measures, CESC remains the leading cause of cancer-related mortality with approximately 270,000 deaths per year [2, 4]. Currently, the primary treatment for CESC patients consists of radiation and/or cisplatin-containing chemotherapy in addition to surgical resection. Unfortunately, the majority of the patients are at an advanced stage that limits therapeutic success when diagnosed. Both local and distant recurrence is common, which highlights the need for improved therapeutic options [5, 6]. The clinical trials of therapeutic HPV vaccines, adoptive T cell therapy, and immune checkpoint inhibitors have shown promising response rates [7–10]. It is nevertheless crucial to identify more effective prognostic biomarkers for CESC and modify the current treatment strategies.

The DNA damage repair (DDR) response maintains genome stability and protects cells against endogenous and exogenous DNA damage [11]. Variations in the DDR genes in tumor cells are frequently associated with high somatic mutation load, which in turn triggers the production of tumor-specific neoantigens [12–15]. Consistently, as reported in a recent study, the DDR gene signature of glioma cells was predictive of patient prognosis and intratumoral immune cell infiltration [16]. Furthermore, the Arg399Gln polymorphism of the X-ray repair cross-complementing group 1 (XRCC1) gene is associated with the prognosis of nonsmall cell lung cancer (NSCLC) patients receiving platinum therapy, and the patients with the Gln/Gln genotype have a survival benefit [17]. Another study reported a correlation between polymorphisms of DDR genes and the response metastatic urothelial cancer patients to PD-1/PD-L1 blockers [18]. Thus, DNA repair defects are potentially novel biomarkers of immune checkpoint blockade response [12]. Mutations in the DNA polymerase required during DNA repair can also improve the overall survival rate of patients by increasing mutations in DDR genes [19]. In addition, mutations in DDR genes are closely related to the resistance of tumors to radiotherapy and chemotherapy [20, 21]. Few studies have reported the clinical significance of DDR genes in CESC, and so far, only XRCC4 has been associated with the progression of cervical cancer [22]. These studies indicate that DDR genes are emerging biomarkers of the clinical prognosis and immunotherapeutic response of various cancers. Furthermore, most DDR genes are regulated by upstream transcription factors (TFs), such as p53, BRCA1, AP-1, and NF-*κ*B [23], which offers new insights into the mechanisms underlying their role in cancer prognosis.

The aim of this study was to identify novel DDR biomarkers for the prognosis and immunotherapeutic response of CESC. To this end, we screened for the differentially expressed DDR genes in CESC from TCGA (The Cancer Genome Atlas) and analyzed their relationship with the immune microenvironment in CESC. A five-DDR gene signature was identified that can predict CESC prognosis and immunotherapeutic response with high sensitivity.

## 2. Materials and Methods

### 2.1. Data Collection

RNA-seq data as well as clinical information (age, days to death, vital status, clinical stage, mutations, copy number variations, etc.) of 306 CESC samples were obtained from TCGA database (https://portal.gdc.cancer). Besides, samples with incomplete clinical information were excluded. DDR gene data was downloaded from AmiGO2. (http://amigo.geneontology.org/).

### 2.2. Identification of Differentially Expressed Genes

The RNA-seq data of DDR genes were processed using the “limma” package. For high-tumor mutation burden (TMB) and low-TMB samples, the differentially expressed genes (DEGs) were screened between them. Univariate Cox regression of the overall survival (OS) was performed using the “survival” R package to identify DDR genes with prognostic relevance.

### 2.3. Identification of Prognostic Genes as well as Establishment of Prognostic Model

Analysis of the prognostic genes was performed by LASSO Cox regression based on the “glmnet” R package. To avoid overfitting, ten-fold cross-validation was adopted to determine the penalized regularization parameter *λ* in the model. For each patient, the risk score was calculated as following: risk score = SUM (expression level of each gene × corresponding coefficient). Based on the median of the risk score, CESC patients were then categorized into the low-/high-risk groups. The Kaplan-Meier curves of both groups were plotted using the “survminer” R package. The “survivalROC” R package was used to plot the time-dependent ROC curve in order to evaluate the predictive power of the gene signature. The independent prognostic predictors of OS were determined by Cox regression using TCGA data. The nomograms and corresponding calibration plots were constructed based on the independent predictors with the “rms” R package, and the predictive power of the nomogram was determined by ROC curve analysis.

### 2.4. Predictors of Immunotherapeutic Response

Single-sample gene set enrichment analysis (ssGSEA) was carried out on thirteen immune-related pathways. Meanwhile, the infiltration of sixteen immune cell types was evaluated using the “gsva” R package. The response of the CESC patients to ICB was predicted on the basis of pretreatment genomics using the tumor immune dysfunction and exclusion (TIDE) program (http://tide.dfci.harvard.edu/).

### 2.5. Identification of the Upstream TFs

TFs coexpressed with the key genes significantly were identified if their correlation coefficients >0.50.

### 2.6. Validation of the Regulatory Mechanism of TFs

For CESC samples, their ATAC-seq data was retrieved from TCGA, and the accessibility of the chromatin located at these biomarker genes were determined. The binding of the TFs to the putative targets was validated by the Cistrome database (http://cistrome.org/db/#/).

### 2.7. Statistical Analysis

Statistical analysis was carried out using R software 4.0.3. For the gene expression levels, Student *t*-test (2-sided) was used to compare the difference of CESC and adjacent nontumor tissues. Besides, Kaplan-Meier method was adopted to evaluate the OS, and log-rank test was used for the comparison between groups. The ssGSEA scores of immune pathways or cells were compared using Mann–Whitney *U* test. *p* < 0.05 indicated statistical significance.

## 3. Results

### 3.1. Identification of Prognostic DDR Genes in CESC

**Figure 1:**
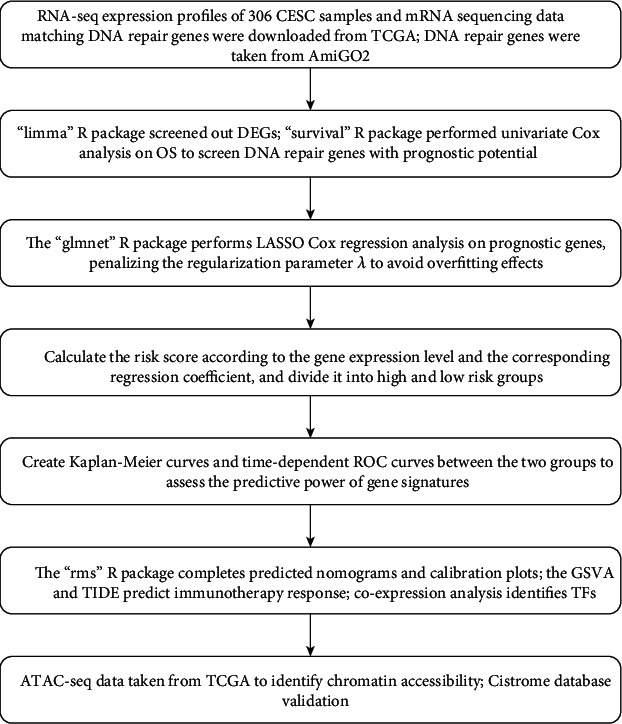
The flow chart of the analysis process. CESC: cervical squamous cell carcinoma; TCGA: The Cancer Genome Atlas; DEGs: differentially expressed genes; OS: overall survival; GSVA: Gene Set Variation Analysis; TIDE: tumor immune dysfunction and exclusion; TFs: transcription factors.

**Figure 2:**
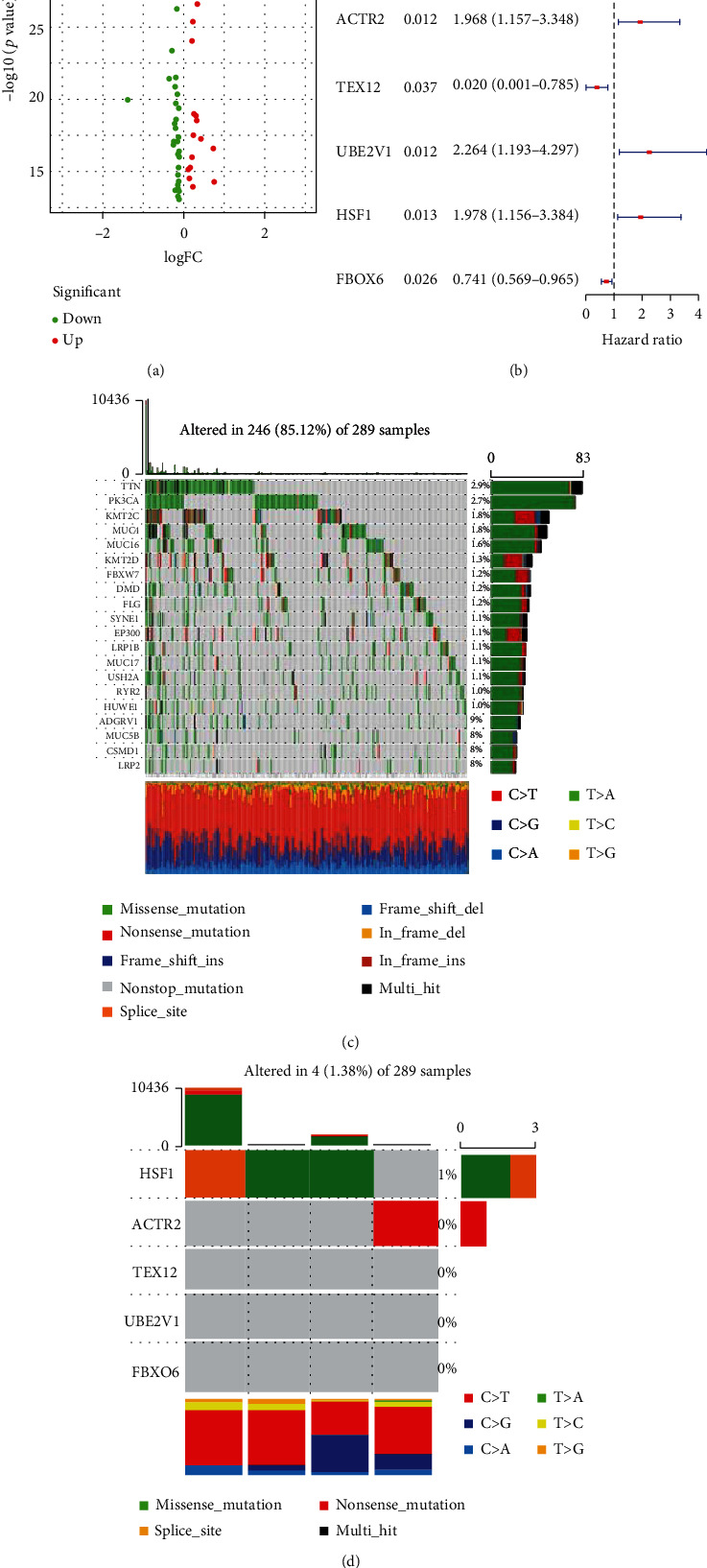
Identification of prognostic genes related to DNA damage repair: (a) Differentially expressed genes in the high and low TMB groups; (b) univariate Cox regression analysis to determine potential prognostic genes; (c) the incidence of major somatic mutations in CESC; (d) the mutation frequency of 5 DNA damage repair genes.

The procedure of bioinformatics analysis is summarized in Figure 1. To identify the prognostic DDR genes, CESC patients were divided into the high and low TMB groups according to the median TMB, and the differentially expressed DDR genes were screened (Figure 2(a)). Univariate Cox regression analysis revealed ACTR2, TEX12, UBE2V1, HSF1, and FBXO6 as the potential prognostic DDR genes (Figure 2(b)). We summarized the incidence of main somatic mutations in CESC (Figure 2(c)) and detected low somatic mutation frequency in the above genes (Figure 2(d)).

### 3.2. Correlation with Prognosis of CESC Patients

**Figure 3:**
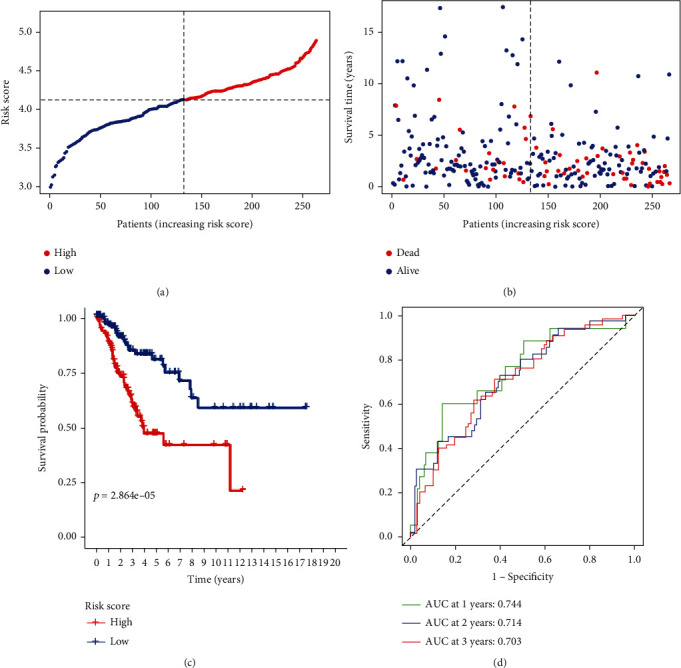
Prognostic analysis of the 5-gene marker in TCGA cohort: (a) risk score of samples from TCGA cohort; (b) the overall survival in TCGA cohort; (c) Kaplan-Meier curves showing the overall survival of the high-/low-risk groups in TCGA cohort; (d) the area under receiver operating characteristic curve showing the prognostic performance of the risk score.

The prognostic model consisting of ACTR2, TEX12, UBE2V1, HSF1, and FBXO6 was established based on LASSO Cox regression. The total risk score of these five genes was calculated as (0.418 × expression of ACTR2) + (−1.995 × expression of TEX12) + (0.147 × expression of UBE2V1) + (0.543 × expression of HSF1) + (−0.217 × expression of FBXO6). Considering the median of the risk score as the cutoff, the samples were categorized into low-/high-risk groups (Figure 3(a)). Compared to the low-risk group, the mortality rate of patients was higher in the high-risk group with statistical significance (Figure 3(b)). Consistently, compared to the high-risk patients, Kaplan-Meier analysis discovered a better OS in low-risk group (Figure 3(c), *p* < 0.05). The areas under the receiver operating characteristic curve (*AUROCs*) for 1-, 2- and 3-year OS were 0.744, 0.714 and 0.703, respectively (Figure 3(d)).

### 3.3. Construction and Verification of DDR-Related Prognostic Model in CESC

**Figure 4:**
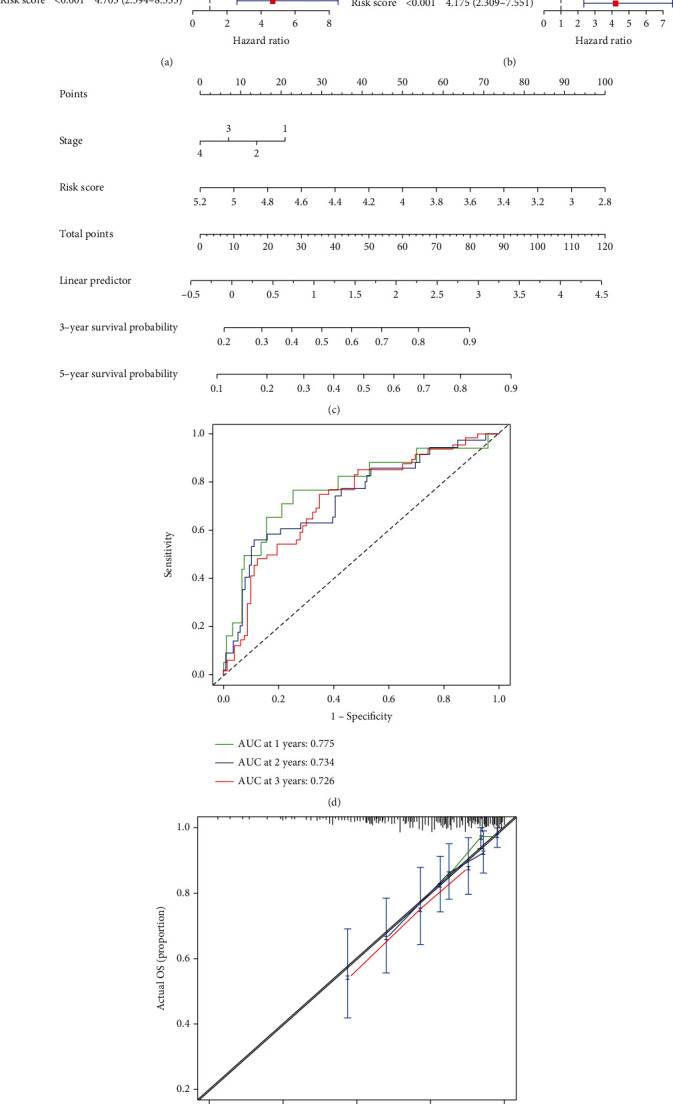
Construction and verification of the prognostic model: (a) the results of the univariate Cox regression analysis for the overall survival in TCGA cohort; (b) the results of multivariate Cox regression analysis; (c) nomogram construction; (d) verification of area under receiver operating characteristic curve.

For the risk score of DDR gene, univariate analysis was used to assess the prognostic value in different subgroups of CESC patients. The risk score and tumor stage were significantly associated with the survival rate of CESC patients, and the risk score had a greater impact. However, no significant difference was observed in the survival rates of patients in terms of age (Figure 4(a)). Multivariate analysis further revealed tumor stage as well as risk score to be prognostic factors for CESC in TCGA cohort (Figure 4(b)). A nomogram consisting of the risk scores and tumor stages was then constructed to put the risk score into clinical prediction (Figure 4(c)). We found that the nomogram could predict the 3- and 5-year OS of cervical cancer patients, and the risk score was the main influencing factor. AUCs were 0.775, 0,734, and 0.726 for 1-, 2-, and 3-year OS, respectively (Figure 4(d)). The prognostic nomogram also showed good predictive ability and clinical value in terms of calibration and decision curve analysis (DCA) (Figure 4(e)). Taken together, the risk score consisting of DDR genes can effectively predict the survival outcomes of CESC patients.

### 3.4. Association between Risk Score and Immunotherapeutic Response of CESC Patients

**Figure 5:**
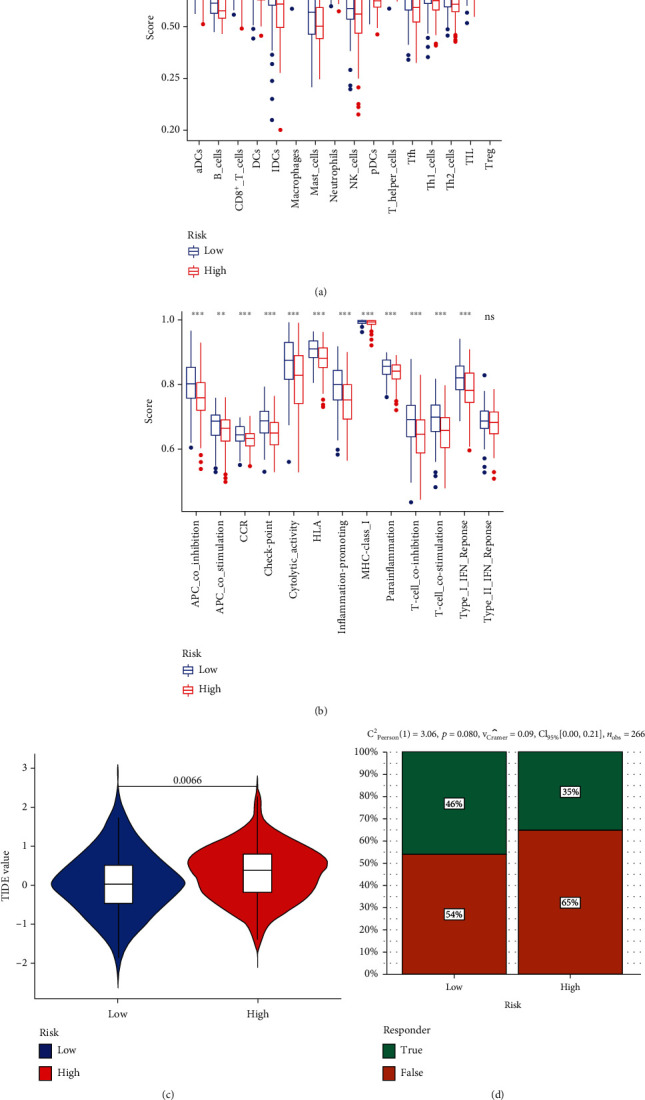
Comparison of ssGSEA scores between low-/high-risk groups in the TCGA cohort: (a) scores for the infiltration of sixteen immune cells; (b) scores for the functional activity of thirteen immune-related pathways; (c) immunotherapy response predicted by TIDE; (d) immunotherapy response rate.

To determine the correlation between risk score and immunotherapeutic response, we quantified the different immune cell subpopulations and activity of immune-related pathways using ssGSEA. Next, the distribution of immune cells and activity score of immune-related pathways between the high-risk group and the low-risk group is used as reliable evidence to assess the infiltration of immune cells. Compared to the low-risk group, the infiltration of 15 immune cell types was lower in the high-risk group with statistical significance, whereas macrophage infiltration showed no difference (Figure 5(a)). Furthermore, all but the type II IFN response immune pathways scored significantly higher in the low-risk group (Figure 5(b)). TIDE results further showed that CESC patients with lower risk score had less immune deficiency (Figure 5(c)) and were able to mount a more potent immune response (Figure 5(d)). In other words, compared to the high-risk patients, those with low-risk scores of DDR gene responded better to immunotherapies. Therefore, the risk score of DDR gene is a reliable biomarker for predicting the immunotherapeutic response in CESC.

### 3.5. FBXO6 Is Downregulated in CESC due to Loss of SP140

**Figure 6:**
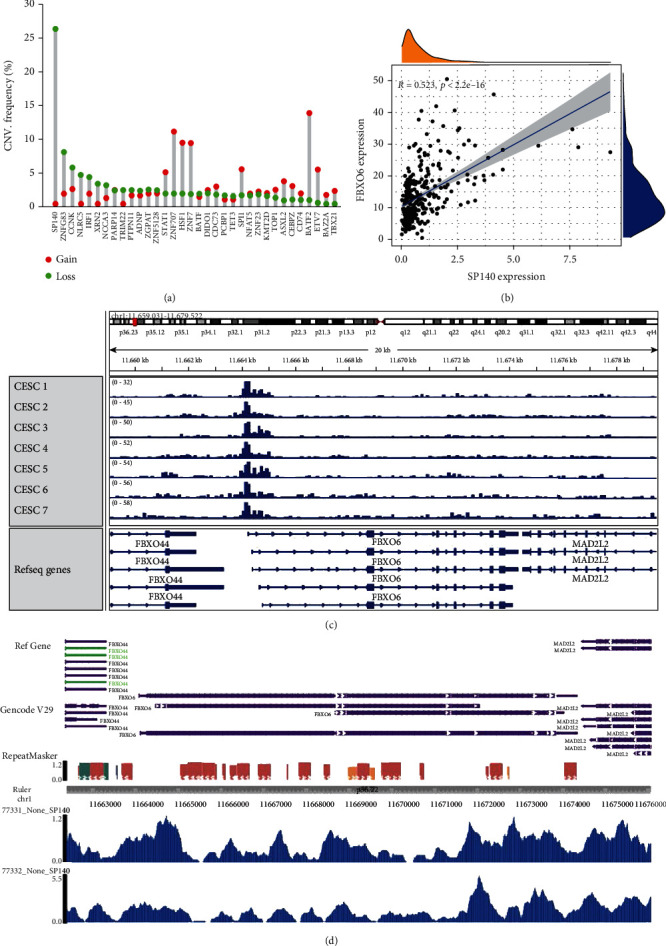
FBXO6 is positively regulated by SP140: (a) volcano map of TFs significantly related to DDR gene, CNV (gene copy number) of SP140 was the least; (b) SP140 is coexpressed with FBXO6; (c) multiple open chromatin regions in the FBXO6 promoter; (d) multiple peaks binding to SP140 in the FBXO6 sequence.

Thirty-five upstream TFs significantly associated with the DDR genes were identified by coexpression analysis, of which SP140 showed maximum copy number loss in CESC (Figure 6(a)). There was clear correlation between the expression levels of SP140 and FBXO6 (*R* = 0.523, *p* < 0.001) (Figure 6(b)). To further explore the underlying mechanisms, we used the ATAC-seq data of SP140 and FBXO6 in CESC samples from TGCA and verified their binding in the Cistrome database. As shown in Figure 6(c), there are multiple open chromatin regions in the promoter of FBXO6, indicating that it is transcriptionally regulated in CESC. We also detected multiple binding peaks corresponding to SP140 in the FBXO6 sequence according to the chip sequence data of SP140 in the Cistrome database (Figure 6(d)). Therefore, the loss of copy number of SP140 in CESC may be a critical factor for FBXO6 downregulation.

## 4. Discussion

DNA carries genetic information which is necessary to synthesize RNA and proteins. Hence, to maintain the structural and functional integrity of DNA is critical for the normal development of all organisms. DNA damage due to endogenous events (oxidative damage, replication fork collapse, or errors that occur naturally during DNA replication or immune cell maturation) or by exogenous factors (ultraviolet rays, ionizing radiation, or chemical reagents) can result in mutations, eventually leading to malignant transformation [24–27]. In order to maintain the integrity of the cellular genome, a series of DNA damage responses, such as repair mechanisms, have evolved that can eliminate or adapt to damage [28].

DDR pathways were consisted of direct repair (DR), base excision repair (BER), nucleotide excision repair (NER), double-strand break repair (DSBR), and interstrand cross-link repair (ICLR) [28] and are regulated by specific genes and their upstream TFs [23]. More than one DDR pathway is often inactivated during cancer initiation and progression, and mutations among DDR genes have been linked to the chemoresistance of tumor cells as well [29, 30]. Thus, DDR genes are prognostically relevant and can be used to predict treatment response along with the overall prognosis of cancer patients. Based on above, we established a prognostic model for CESC involving five DDR genes, which accurately predicted the survival, immune infiltration, and the efficacy of immunotherapy in CESC patients. Thus, this novel prognostic signature can be used to select suitable patients for immunotherapy.

Referring to the expression levels of the five DDR genes, CESC patients are divided into low-/high-risk groups, and the former exhibited worse prognosis in terms of the OS rates. The TNM staging and risk scores of the low-/high-risk groups were significantly different, whereas age did not have a significant impact on the prognosis. Furthermore, the risk score was identified as an independent prognostic factor. The nomogram indicated high predictive power of the risk score for 3- and 5-year OS, whereas ROC analysis showed that 1-, 2-, and 3-year OS could be predicted by the risk score. The accuracy of this prognostic model was also validated by the decision curve analysis (DCA). Thus, the DDR gene-based risk score can precisely forecast the survival outcome of CESC patients and, at the same time, provide more therapeutic options.

Interestingly, for the low-/high-risk groups, the infiltration of 15 immune cell types differed significantly, whereas the infiltration of macrophages was similar. The low-risk group showed greater immune cell infiltration, especially that of T-helper cells, Treg cells, and CD8+ T cells. Furthermore, 13 immune-related pathways scored higher in the low-risk group, while type II IFN response showed no significant difference between groups. In addition, the association between the risk score of CESC and immunotherapeutic response was evaluated by TIDE program and revealed 46% and 35% positive responders in the low-risk and high-risk groups, respectively, which further underscores the role of DDR genes in determining the response of CESC patients to immunotherapy.

The checkpoint kinase CHK1 (CHEK1) recognizes DNA damage, delays the cell cycle, and initiates DNA repair [31]. FBXO6 can specifically recognize activated CHEK1 and promote its ubiquitin-dependent degradation [32], thereby inhibiting DNA repair and function and eventually leading to increased neoantigen diversity and sensitivity to immunotherapy. Studies show that FBXO6 expression is related to the OS of NSCLC patients, and *in vitro* experiments have shown that FBXO6 inhibits cell proliferation, promotes apoptosis, and sensitizes the cells to cisplatin [33]. Furthermore, FBXO6 also inhibits the antiviral response by interfering with the production of IFN-I [34]. Ji et al. reported that the high expression levels of FBXO6 in tissues were correlated with poor survival of patients with advanced ovarian cancer. FBXO6 directly interacts with the tumor suppressor gene RNASET2 to target it for ubiquitin-dependent degradation, thus functioning as an oncogene in ovarian cancer [35]. Wang et al. found that FBXO6 is one of the coexpressed genes on CD8+ T cells and promotes infiltration of the cells into urothelial carcinoma tumors, which affects the clinical phenotype and the immune microenvironment [36].

TFs are the main regulators of gene expression in eukaryotic cells [37]. SP140 belongs to the speck protein (SP) family of TFs that are also known as human chromatin “readers.” A chromatin reader is the core interpreter of the epigenome that promotes cell-specific transcription and is a therapeutic target for cancer and inflammation [38, 39]. SP140 is involved in various immune-related diseases such as Crohn's disease, chronic lymphocytic leukemia, and multiple sclerosis [40–42] and has recently been identified as the main regulator of the immune response in ovarian cancer [43]. We detected a significant decrease in the copy number of SP140 in CESC patients, which correlated with the downregulation of its downstream target FBX06.

**Figure 7:**
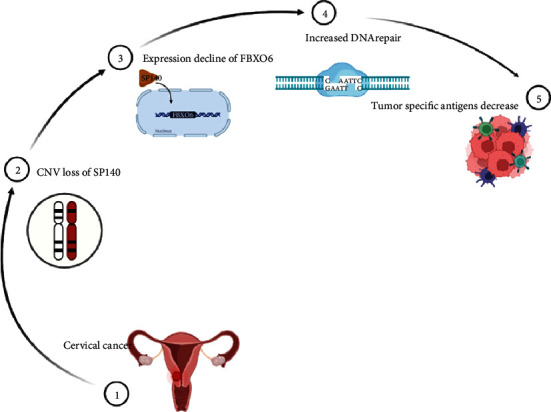
Schematic representation of the mechanism of FBXO6 and SP140 in CESC.

Our study is the first to show that SP140-FBXO6 is related to the prognosis as well as immune microenvironment of CESC. Loss of SP140 in CESC cells downregulated the DNA repair gene FBXO6, which resulted an increased DNA repair and decreased generation of tumor-specific antigens (Figure 7). Thus, DDR genes are promising biomarkers of prognosis and immunotherapeutic response of CESC. Further studies are necessary to elucidate mechanism of SP140/FBXO6 in cervical cancer. In addition, it will be challenging to combine the DDR gene status with other known biomarkers for clinical applications.

## 5. Conclusion

We identified five DDR genes that are related to the OS of CESC patients, and the gene signature can predict prognosis and the response to immunotherapy. Nevertheless, the markers will have to be verified by functional analyses and clinical tests, and further research on the molecular mechanism of these genes is urgently needed.

## Data Availability

The datasets generated during and/or analyzed during the current study are available from the corresponding author on reasonable request.
